# Characterization of Learning, Motivation, and Visual Perception in Five Transgenic Mouse Lines Expressing GCaMP in Distinct Cell Populations

**DOI:** 10.3389/fnbeh.2020.00104

**Published:** 2020-06-23

**Authors:** Peter A. Groblewski, Douglas R. Ollerenshaw, Justin T. Kiggins, Marina E. Garrett, Chris Mochizuki, Linzy Casal, Sissy Cross, Kyla Mace, Jackie Swapp, Sahar Manavi, Derric Williams, Stefan Mihalas, Shawn R. Olsen

**Affiliations:** Allen Institute for Brain Science, Seattle, WA, United States

**Keywords:** mouse behavior, GCaMP transgenic mice, visual perception, motivation, learning, change detection

## Abstract

To study the mechanisms of perception and cognition, neural measurements must be made during behavior. A goal of the *Allen Brain Observatory* is to map the activity of distinct cortical cell classes underlying visual and behavioral processing. Here we describe standardized methodology for training head-fixed mice on a visual change detection task, and we use our paradigm to characterize learning and behavior of five GCaMP6-expressing transgenic lines. We used automated training procedures to facilitate comparisons across mice. Training times varied, but most transgenic mice learned the behavioral task. Motivation levels also varied across mice. To compare mice in similar motivational states we subdivided sessions into over-, under-, and optimally motivated periods. When motivated, the pattern of perceptual decisions were highly correlated across transgenic lines, although overall performance (d-prime) was lower in one line labeling somatostatin inhibitory cells. These results provide important context for using these mice to map neural activity underlying perception and behavior.

## Introduction

Goal-oriented behavior involves coordinated neural activity across brain regions, but the cellular mechanisms mediating these activity dynamics are not fully understood. The mouse provides unique opportunities to dissect cell type- and circuit-specific mechanisms of perception and behavior (Luo et al., 2008, 2018; Niell, 2015). Head-fixed behaviors are well-established and allow precise measurements of cellular activity using 2-photon imaging and electrode recordings, in addition to optogenetic perturbations (Andermann et al., 2010; O’Connor et al., 2010; Histed et al., 2012; Guo et al., 2014b; Burgess et al., 2017). Applications of these methods are revealing mechanisms of perception and action across multiple sensory modalities and cognitive systems (Harvey et al., 2012; Huber et al., 2012; Petreanu et al., 2012; Chen et al., 2013; Glickfeld et al., 2013; O’Connor et al., 2013; Pinto et al., 2013; Guo et al., 2014a; Peron et al., 2015; Poort et al., 2015; Goard et al., 2016; Li et al., 2016; Resulaj et al., 2018).

At the Allen Institute we seek to generate a database of cell type-specific activity across visual cortical areas during visual stimulation and behavior (Koch and Reid, 2012). We previously developed a standardized physiological pipeline—the *Allen Brain Observatory—* to monitor cellular population activity using 2-photon calcium imaging during passive visual stimulation in mice (De Vries et al., 2020). These experiments used transgenic Cre driver mouse lines to express the genetically encoded calcium indicator GCaMP6 in specific cortical cell subpopulations to monitor activity based on changes in cellular fluorescence. To expand on these passive viewing datasets, we are adapting our existing pipeline to include GCaMP6 measurements from mice performing visually guided behaviors. For large-scale pipeline compatibility we seek tasks that are simple yet adaptable to more complex variants, easily learned, and consistently performed. Candidate tasks must also support head-fixed physiological measurements using our standardized instruments.

In this study we test a go/no-go visual change detection task. Change detection is a fundamental behavioral capacity of animals and humans (Rensink, 2002; Elmore et al., 2011; Hagmann and Cook, 2013; Pearson and Platt, 2013), and the visual cortex of mice and primates is implicated in the detection of changes in visual features (Womelsdorf et al., 2006; Glickfeld et al., 2013; Brunet et al., 2014). The core task we describe can be used to test perception of various visual features including orientation, contrast, color, and natural images (Glickfeld et al., 2013; Denman et al., 2018; Garrett et al., 2020). Moreover, our task includes features that permit investigation of the physiological correlates of behavior and cognition. For instance, this task allows for exploration of stimulus novelty and learning, temporal expectation (Garrett et al., 2020), and short-term memory (Hu et al., 2020).

To support future studies of neural activity during this task, we have characterized learning and behavior of five Cre driver × GCaMP6 reporter transgenic mouse lines, each of which expresses the GCaMP6 calcium sensor in distinct subpopulations of excitatory or inhibitory cells of the neocortex (Madisen et al., 2015; De Vries et al., 2020; Garrett et al., 2020). These cortical cell subpopulations are believed to play distinct functional roles in cortical computation (Kepecs and Fishell, 2014; Harris and Shepherd, 2015). We test a Cux2-Cre driver line labeling excitatory cells in layers 2, 3, and 4 of the cortex that allows measurement of activity in superficial cortical neurons. We test a Rbp4-Cre driver line that labels neurons in cortical layer 5, which is the major subcortically projecting layer. The third excitatory line we test is Slc17a7-Cre, which is a pan-excitatory line that results in GCaMP6 expression in all excitatory neurons of the cortex. Because GABAergic inhibitory cells are critical for local circuit function in the cortex, we also tested two inhibitory lines that label two of the major inhibitory cell subclasses of the cortex. First, we test a Vip-Cre driver line which labels the vasoactive intestinal polypeptide-expressing (Vip) inhibitory neurons in the cortex. Second, we test a Sst-Cre driver line which labels the somatostatin-expressing (Sst) subpopulation of inhibitory neurons of cortex. To mitigate sources of variability in behavior and facilitate comparisons across mice from these five transgenic lines we used automated training procedures in this study.

## Materials and Methods

### Mice

All experiments and procedures were performed in accordance with protocols approved by the Allen Institute Animal Care and Use Committee. Male and female transgenic mice expressing GCaMP6 in various Cre-defined cell populations were used in these experiments (Madisen et al., 2015). The five genotypes used in this study were *Cux2*: Cux2-CreERT2;Camk2a-tTA;Ai93(TITL-GCaMP6f), *n* = 4; *Rbp4*: Rbp4-Cre_KL100;Camk2a-tTA;Ai93(TITL-GCaMP6f), *n* = 12; *Slc17a7*: Slc17a7-IRES2-Cre;Camk2a-tTA;Ai93(TITL-GCaMP6f), *n* = 23; *Sst*: Sst-IRES-Cre;Ai148(TIT2L-GC6f-ICL-tTA2), *n* = 7; *Vip*: Vip-IRES-Cre;Ai148(TIT2L-GC6f-ICL-tTA2), *n* = 14. Prior to surgery mice were singly housed and maintained on a reverse 12-h light cycle (off at 9 am, on at 9 pm); all experiments were performed during the dark cycle. The set of mice used in these experiments are shown in Supplementary Table 1.

### Surgery

Some of the mice included in this study were later used in a set of 2-photon calcium imaging experiments (Garrett et al., 2020) and, as such, they all initially received a headpost and cranial window surgery as previously described (De Vries et al., 2020; Groblewski et al., 2020a). Briefly, surgery was performed on healthy mice that ranged in age from 5 to 12 weeks. Mice were deeply anesthetized with isoflurane prior to removing skin and exposing the skull. A custom titanium headframe was cemented to the skull and a circular piece of skull 5 mm in diameter was removed, durotomy performed, and a glass coverslip stack was cemented in place. Upon successful recovery from surgery mice entered into behavioral training.

### Behavior Training

#### Water Restriction and Habituation

Throughout training mice were water-restricted to motivate learning and performance of the behavioral task (Guo et al., 2014b). Mice had access to water only during behavioral training sessions or when provided by a technician on non-training days. During the first week of water restriction mice were habituated to daily handling and increasing durations of head fixation in the behavior enclosure over a 5-day period. The first day of behavior training began after 10 days of water restriction. Mice were trained 5 days per week (Monday–Friday) and were allowed to earn unlimited water during the daily 1 h sessions; supplements were provided in a home cage water dish if the earned volume fell below 1.0 mL and/or body weight fell under 80–85% of initial baseline weight. On non-training days mice were weighed and received water provision to reach their target weight, but never less than 1.0 mL per day.

#### Apparatus

Mice were trained in custom-designed, sound-attenuating behavior enclosures equipped with a 24′′ gamma-corrected LCD monitor (ASUS, #PA248Q). Mice were head-fixed on a behavior stage with 6.5′′ running wheel tilted upwards by 10–15 degrees. The center of the visual monitor was placed 15 cm from the eye and visual stimuli were spherically warped to account for the variable distance from the eye toward the periphery of the monitor. Water rewards were delivered using a solenoid (NI Research, #161K011) to deliver a calibrated volume of fluid through a blunted, 17 g hypodermic needle (Hamilton) positioned approximately 2–3 mm away from the animal’s mouth.

#### Change Detection Task

##### Overview

**FIGURE 1:**
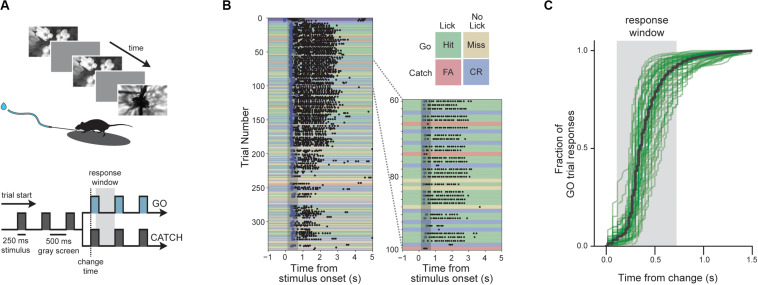
Change detection task with natural images. **(A)** Behavioral task. Visual stimuli are shown for 250 ms with an intervening gray period of 500 ms. On GO trials, the image identity changes and mice must lick within the 600 ms response window to receive a water reward. On CATCH trials, no image change occurs, and licking is measured to quantify guessing behavior. **(B)** Example of a complete behavior session with trials aligned to the time of image change. Blue dots indicate rewards and black dots indicate licks. Trial types and outcomes are illustrated in the 2 × 2 matrix. **(C)** Cumulative reaction time distribution on GO trials. Green lines show individual mice (*n* = 56) with trials pooled across all included sessions, and the black line indicates the average of all mice.

Mice were trained for 1 h/day, 5 days/week using a behavioral program implementing a go/no-go change detection task schematized in Figure 1. Briefly, mice were trained to lick a reward spout when the identity of a flashed visual stimulus changed identify. If mice responded correctly within a short, post-change response window (115–715 ms) a water reward was delivered. The volume of contingent rewards was 10 μL in Stages 1 and 2, and reduced to 7 μL after the first 3 sessions of Stage 3. The four stages of the training protocol are shown below:

**Table:** 

**Stage**	**Stimulus**	**Stimulus Presentation**	**Response Window (ms)**	**Contingent Rewards**	**Duration (min)**
**0**	Square-wave gratings	Static	NA	False	15
**1**	Square-wave gratings	Static	1000	True	60
**2**	Square-wave gratings	250 ms stimulus; 500 ms gray period	600	True	60
**3**	Natural Images	250 ms stimulus; 500 ms gray period	600	True	60
					

On Day 1 of the automated training protocol mice received a short, 15-min “open loop” session during which non-contingent water rewards were delivered coincident with 90°changes in orientation of a full-field, static square-wave grating (Stage 0). This session was intended to (1) introduce the mouse to the fluid delivery system and, (2) provide the technician an opportunity to identify the optimal lick spout position for each mouse. Each session thereafter was run in “closed loop,” and progressed through 3 phases of the operant task: (1) static, full-field square wave gratings (oriented at 0° and 90°, with the black/white transition always centered on the screen and the phase chosen randomly on every trial), (2) flashed, full-field square-wave gratings (0° and 90°, with phase as described in (1), and (3) flashed full-field natural scenes (eight natural images used in the *Allen Brain Observatory*^1^).

##### Progression through training stages

Starting with Stage 1, the advancement criteria required mice to achieve a session maximum performance of at least d-prime = 2 (calculated over a rolling 100 trial window without trial count correction) during two of the last 3 sessions. The fastest progression from Stage 1 to Stage 3 was 4 training days.

##### Behavior session and trial structure

Each behavior session consisted of a continuous series of trials, schematized in Supplementary Figure 1A. Briefly, prior to the start of each trial a trial-type and change-time were selected. Trial-type was chosen based on predetermined frequencies such that “GO” and “CATCH” trials occurred with specified probabilities. In stages 1 and 2, the catch probability was set at 25%, but no more than three consecutive trials of a given type were permitted, leading to an effective catch probability of ∼36%. In stage 3, the catch probability was initially set at 12.5% (given that the 8 same-to-same changes represented 8/64 possible image changes), which, combined with the maximum of 3 consecutive go/catch trial rule, led to an effective catch probability of ∼30%. However, later sessions implemented a matrix sampling algorithm that ensured that each image transition was sampled equally, pushing the actual catch probability to ∼12.5%. Change-times were selected from a truncated exponential distribution ranging from 2.25 to 8.25 s (mean of 4.25 s) following the start of a trial. Due to computational lag when aligning change-time with a stimulus flash, the actual distribution of change times was shifted to the right by one 750 ms flash cycle (with only a small fraction of changes occurring at 2.25 s) resulting in a mean change time of 4.2 s. In trials when a mouse licked prior to the stimulus change the trial was reset, and a timeout period was imposed. The number of times a trial could be reset before re-drawing the timing parameter was limited to five. In all, this trial structure leads to a sampling of “GO” and “CATCH” trials, that when combined with mouse responding, yields “HIT,” “MISS,” “FALSE ALARM,” and “CORRECT REJECTION” trials.

In addition to the four trial types described above, behavior sessions contained a subset of “free reward” trials (“GO” trials followed immediately by delivery of a non-contingent reward). Behavior sessions across all phases began with 5 “free-reward” trials. Additionally, in order to promote continued task performance throughout the behavior session “free reward” trials were delivered after 10 consecutive “MISS” trials. All non-contingent rewards were 5 μL in volume.

### Data Analysis

Analysis was performed using custom scripts written in Python v3.7.5 (including Pandas v0.24.2, Numpy v1.16.4, Scipy v1.3.2 and Statsmodels v0.10.1) and GraphPad Prism (v8.0.1). Plots were generated using Matplotlib v3.1.1 and Seaborn v0.9.0.

Behavioral performance was quantified with the signal detection metrics of d-prime and criterion, which are both a function of hit and false alarm rates.

#### Hit and False Alarm Rates

The hit rate was calculated as the fraction of go-trials in which the mouse licked in a 0.115 to 0.715 s window following the display-lag-compensated image display time. Catch trials were defined as trials in which there was no image change. However, for calculation of the false alarm rate, a response window was defined following one of the flashes using the same statistics as in the go trials. False alarm rates were calculated as the fraction of catch-trials in which animal emitted a lick in this response window. Unless otherwise noted, hit and false alarm rates were corrected to account for trial counts using the following formula (Macmillan and Creelman, 2004):

(1) 1/(2N)⁢<=HR<=(1-1/(2⁢N))

(2) 1/(2⁢N)<=FAR<=(1-1/(2⁢N))

Where HR and FAR represent the hit and false alarm rates, and N represents the number of the respective trial type.

#### D-Prime (d’)

D-prime, which is a measure of the relative difference in response probabilities across the two trial types, is defined as:

(3) d-prime=Z⁢(HR)-Z⁢(FAR)

in which Z represents the inverse cumulative normal distribution function.

#### Criterion

Criterion, which is a measure of the underlying bias of the subject to emit a response, is defined as:

(4) C=-1/2⁢[Z⁢(HR)+Z⁢(FAR)]

Criterion therefore varies from negative values for high response biases (high hit and false alarm rates) to positive numbers for low response biases (low hit and false alarm rates). In general, our figures represent criterion with the sign inverted, thus mapping states of low motivation to negative values and states of high motivation to positive values.

### Statistical Analysis

Statistical comparisons between multiple groups were performed using both parametric (ANOVA) and non-parametric (Kruskal–Wallis) tests with *post hoc*, pairwise comparisons corrected for multiple comparisons. Independent pairwise comparisons were made using *t*-tests and Wilcoxon signed-rank tests. Correlational analyses were performed using Pearson correlation coefficients.

We used a bootstrap analysis to assess statistical differences in d-prime values. Bootstrapping involved subsampling with replacement, with sample size determined by the group with the smallest value count. One thousand bootstrap iterations were performed. Comparisons of bootstrapped distributions were performed by calculating the total density of the joint probability distribution on one side of the unity line, yielding a probability, *p*_boot_, that null hypothesis is true (Saravanan et al., 2019). Pairwise comparisons were deemed significant if the fraction of overlap was less than the Bonferroni corrected two-tailed alpha. The resolution of *p*_boot_ was limited by the number of bootstrap iterations (1000), providing a minimum measurable value of 0.001.

## Results

### Visual Change Detection Task With Natural Scene Images

We trained mice (*n* = 60) to perform a visual change detection task with natural scene images. In this go/no-go task, mice see a continuous series of briefly presented images and they earn water rewards by correctly reporting when the identity changes (Figure 1). Responses are indicated by licking a water spout within a 600 ms response window following the image change (Figures 1A,B). On randomly interleaved ‘catch’ trials, no image change occurs and the mouse must withhold licking to avoid a time-out (Figures 1A,B). Once trained, mice display short latency reaction times with the majority of responses occurring within the response window (Figure 1C).

In our behavioral apparatus, mice are head-fixed yet free to run on a circular disk. Running is monitored but does not influence task flow. Most, but not all, mice ran or walked during the behavioral session, and these mice typically stopped running when responding to stimulus changes and to consume the water reward (Supplementary Figure 2).

### Automated Behavior Training of Transgenic Mice

**FIGURE 2:**
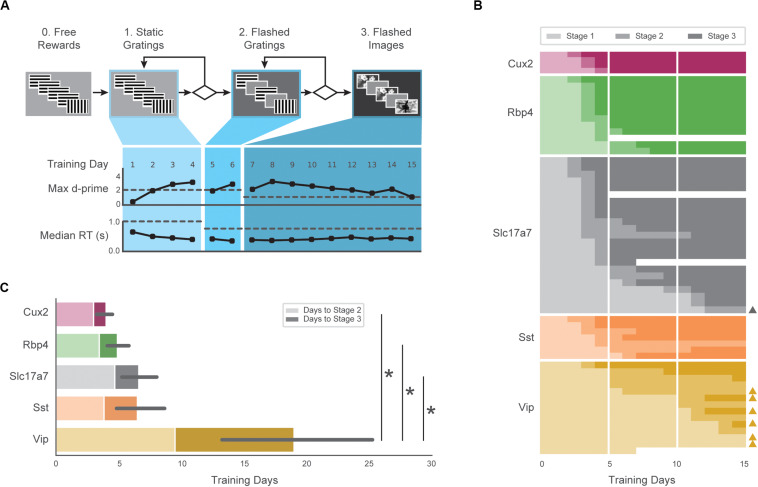
Automated training of five GCaMP6-expressing transgenic lines. **(A)** Top: Progression of training stages. Bottom: Training trajectory for one example mouse (M328341, genotype: Rbp4). Max d-prime (in 100-trial rolling window without trial count correction) and median reaction time are shown for each training day. Horizontal dashed lines represent the max d-prime required for advancement and the maximum reaction time following stimulus change which would result in reward. **(B)** Training days in each stage (each row is one mouse) for first 15 days of training. Opacity of bar indicates training stages 1–3 in **(A)**. Triangles on right indicate mice that reached Stage 3 after 3 weeks of training. Some mice (*n* = 4) were removed from training early due to a health-related issue (designated with white). **(C)** Average number of sessions required to reach Stage 2 (light shading) and Stage 3 (dark shading) for all groups. Non-parametric analysis showed a significant main effect of group on time to Stage 3, with Vip mice exhibiting significantly longer training times than Slc17a7, Rbp4, and Cux2 mice. Error bars represent the 95% bootstrapped confidence interval for all mice that reached stage 3 in each genotype.

We assessed training and performance of five transgenic mouse lines expressing GCaMP6f in distinct subsets of cortical cells [*Cux2*: Cux2-CreERT2;Camk2a-tTA;Ai93(TITL-GCaMP6f); *Rbp4*:Rbp4-Cre_KL100;Camk2a-tTA;Ai93(TITL-G CaMP6f); *Slc17a7*: Slc17a7-IRES2-Cre;Camk2a-tTA;Ai93(TITL-GCaMP6f); *Sst*: Sst-IRES-Cre;Ai148(TIT2L-GC6f-ICL-tTA2); *Vip*: Vip-IRES-Cre;Ai148(TIT2L-GC6f-ICL-tTA2)]. To train these transgenic mice (Cux2, Rbp4, Slc17a7, Sst, Vip) in a standardized manner, we developed an automated protocol in which mice progress through a series of training stages with parameters, performance requirements, and stage transitions defined in software rather than relying on experimenter intervention (Figure 2A).

The majority of mice (47/60) completed the full set of training stages within 15 sessions, and 56/60 mice reached the final stage within 40 sessions (Figure 2B). The average time to reach the final training stage varied across genotypes (Figure 2C; Cux2, 4.0 ± 0.8; Rbp4, 4.9 ± 1.4; Slc 6.6 ± 3.5; Sst, 6.5 ± 2.6, Vip, 19.0 ± 10.9), and there was a significant main effect of genotype on training times (*H* = 22.98, *p* = 0.0001). *Post hoc*, pairwise comparisons showed Vip transgenic mice were slower to train than the Slc (*p* = 0.0002), Rbp4 (*p* = 0.0005), and Cux2 groups (*p* = 0.003). Thus, all genotypes were able to learn the task, but the number of sessions to do so varied.

All subsequent data analysis is restricted to sessions in the final training stage (stage 3) in which mice had peak hit rate and d-prime values (both calculated over a rolling 100 trial window) of at least 0.3 and 1.0, respectively, and had at least 50 correct responses on hit trials. Of 1319 sessions in the final training stage, 1100 met these performance criteria. Of the 60 mice in the study, 56 mice had at least one included stage 3 session. Supplementary Table 1 provides a detailed summary of the mice described in this study, including the number of sessions analyzed.

### Variation in Motivation

**FIGURE 3:**
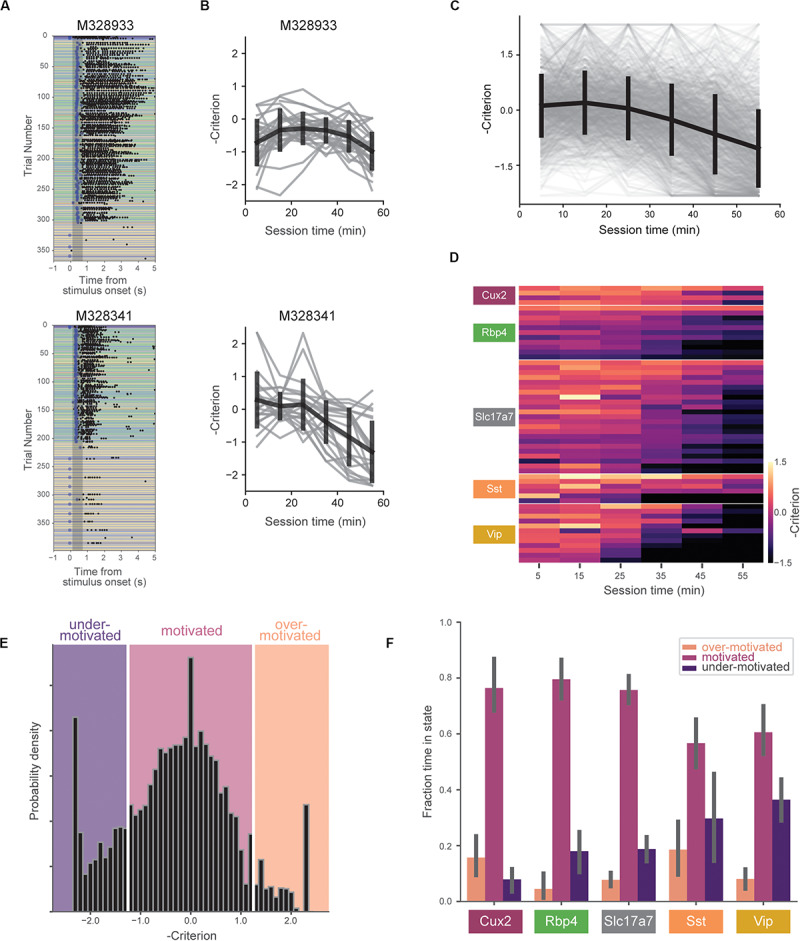
Motivation decreases over behavioral session. **(A)** Example behavioral sessions from two mice showing high task-engagement early in the session followed by later disengagement. **(B)** Negative criterion (0.5*[z(HR) + z(FA)]) computed in 10-min bins for same mice in **(A)**. Individual sessions are shown in gray and the mean over all sessions (±SD) is shown in black. Note the sign inversion to map states of low engagement to negative numbers and *vice-versa*. **(C)** Criterion in 10-min bins for all sessions (gray) and mean (±SD) across all mice (black). **(D)** Across-session average of criterion values for all mice (each row represents a single mouse). Rows are sorted by genotype and average criterion value. **(E)** Histogram of negative criterion values (10-min epochs, again note the sign inversion) for all sessions. White lines indicate boundaries for defining three motivation states: ‘over-motivated’ (criterion > 1.25, 6.9%), ‘motivated’ (–1.25 ≤ criterion ≤ 1.25, 73.2%), and ‘under-motivated’ (criterion < –1.25, 18.6%). Epochs without at least one hit trial and one false alarm trial (1.2% of the total) were not assigned a criterion value (and thus not included). **(F)** Fraction of session epochs spent in each engagement state. Error bars represent bootstrapped 95% confidence intervals. All groups except Sst and Vip groups spent significantly more time in motivated versus under-motivated states.

In typical behavior sessions, mice were very responsive early but became less task-engaged later in the hour-long session. During these periods of reduced task-engagement, mice licked only infrequently, or ceased licking altogether, indicating that motivation to perform the task decreased (Figure 3A).

We quantified changes in motivation using the ‘criterion’ parameter from signal detection theory (−0.5^∗^[z(HR) + z(FA)]). Criterion is a measure of the subject’s internal bias to respond. Higher values correspond to more conservative response criteria and correspondingly lower response rates. To aid visualizations we represent criterion with the sign inverted, thus mapping states of low motivation to lower values and states of high motivation to higher values. To capture motivation changes over the course of the behavioral session, we computed criterion in 10-min epochs. On average, mice showed decreasing motivation over the course of the 1-h session (Figures 3B,C), but we observed a range of motivation levels across mice and genotypes (Figure 3D).

To compare mouse behavior during similar motivational states, we subdivided behavioral sessions into epochs labeled ‘over motivated’ (criterion > 1.25), ‘motivated’ (−1.25 ≤ criterion ≤ 1.25), and ‘under motivated’ (criterion < -1.25) (Figure 3E). Over motivated states are characterized by periods in which the mice have very high response rates for both GO and CATCH trials, whereas under motivated states are characterized by low rates of response for both these trials. A small percentage of epochs (1.2%) were not assigned a criterion value due to insufficient presentations of GO and/or CATCH trials in 10-min epoch (Supplementary Table 1). Mice spent the majority of their time in the ‘motivated’ state (Figure 3F), however, there was a significant interaction between genotype and state [*F*(8,102) = 4.87, *p* < 0.0001]. Follow-up, within-genotype pairwise comparisons indicated that all but the Vip and Sst groups spent significantly more time in the motivated state than in the under-motivated state (*p* < 0.01 for comparisons in Cux2, Rbp4, and Slc17a7 groups).

**FIGURE 4:**
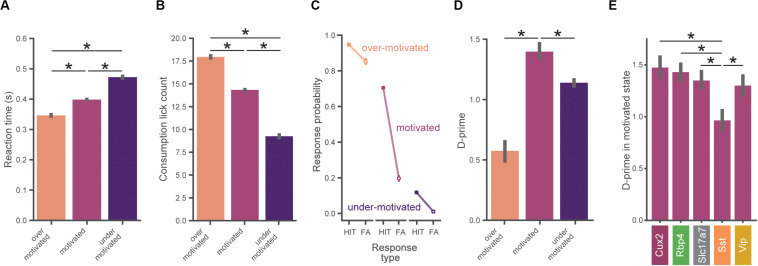
Task performance varies across motivation states. **(A)** Reaction times are slower with lower motivation. In **(A–D)**, data is pooled across all mice (*n* = 56) and motivation state is defined as in Figure 3E. All error bars represent bootstrapped 95% confidence intervals. **(B)** Total number of water consumption licks is less with lower motivation. Total licks are counted in a 5 s window following reward. **(C)** Hit and false alarm rates in each motivation state (defined as in Figure 3E). **(D)** Inverted-U relationship between d-prime and motivational level. D-prime is higher in motivated compared to over- and under-motivated states. **(E)** D-prime in the motivated state for each genotype. The Sst group exhibited a lower d-prime than each of the other genotypes in the motivated state. ^∗^Indicates significance using a Bonferroni corrected *p*-value of 0.05/N_comparisons.

The consistent progression from over-motivation to under-motivation likely reflects waning engagement due to decreasing thirst in the session. Supporting this, licking reaction times (pooled across mice) were shortest when mice were over-motivated but longest when under-motivated (Figure 4A, *H* = 6632.17, *p* < 0.0001; *p* < 0.001 for all pairwise comparisons). Additionally, consumption lick counts (the number of licks in a 5 s window following reward delivery, which is a metric of response vigor) were highest when mice were over-motivated but lowest when under-motivated (Figure 4B, *H* = 5349.45, *p* < 0.0001; *p* < 0.001 for all pairwise comparisons) (Berditchevskaia et al., 2016).

### Behavioral Performance Varies With Motivation

The probability of a behavioral response (averaged over all images) varied with motivation levels, as expected from our criterion-based definition (Figure 4C). When over-motivated, both hit and false alarm rates were high. In the more optimal motivational range, hit rates were high but false alarm rates were low. Finally, when under-motivated, mice showed low hit and false alarm rates.

To assess psychophysical performance for each motivational state we computed d-prime values by pooling across all trials from all mice in matched motivational states in order to reduce the impact of epochs with low trial counts (which would provide less accurate estimates of d-prime). We found an inverted-U shape relationship between d-prime and motivation level (Figure 4D), consistent with both classic (Yerkes and Dodson, 1908; Duffy, 1957) and recent studies (Mcginley et al., 2015a). We performed a series of pairwise hypothesis tests on the bootstrapped d-prime distributions and found that d-prime was greater in the motivated state than in both the under- and over-motivated states (*p*_boot_ < 0.001). Thus, periods of ‘optimal’ motivation corresponded to the highest performance as measured with d-prime. Supplementary Figure 3 illustrates how the relationship of d-prime and motivation varies with different criterion thresholds for defining motivational states.

We next computed d-prime values in the motivated state separately for each genotype using the same bootstrap analysis described above. Motivated d-prime values were not significantly different across genotypes, except for the Sst group which had a lower d-prime compared to each of the other groups (Figure 4E, *p*_boot_ < 0.001). Despite our efforts to include both males and females in this study, sex was not evenly matched across the groups (both the Cux2 and Sst groups were all male, see Supplementary Table 1), therefore we repeated the between-genotype analysis using only male mice (*n* = 34). With analysis restricted to male mice only, d-prime in the Sst group remained significantly lower than all other groups (*p*_boot_ < 0.001), with no other groups showing significant differences.

### Highly Correlated Perception Across Transgenic Lines in Motivated State

**FIGURE 5:**
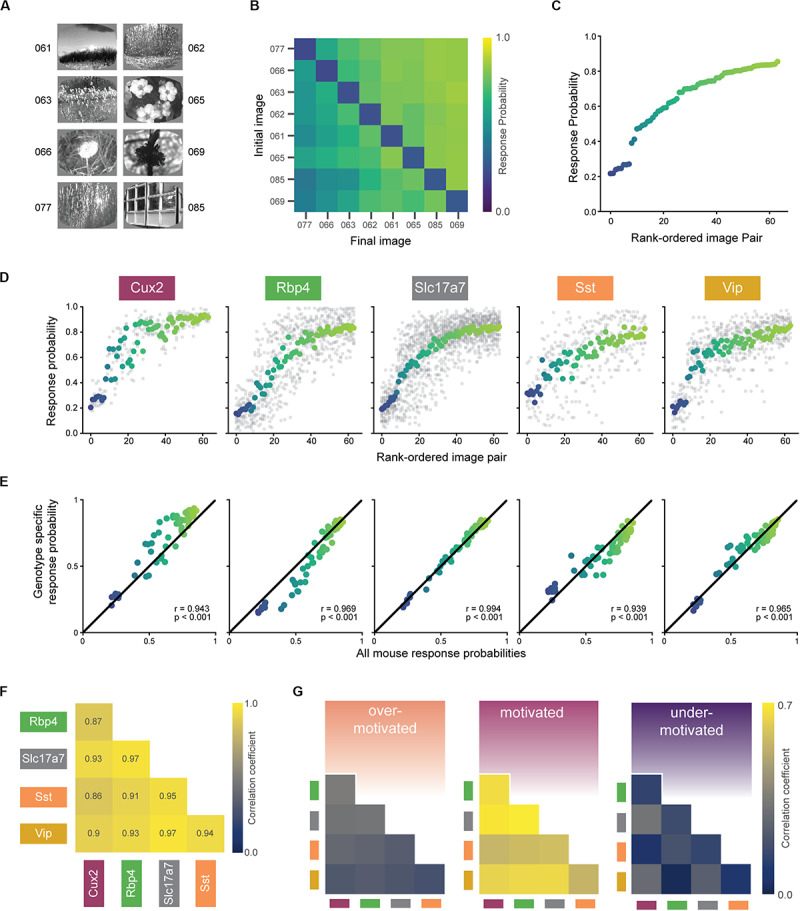
Similar perception across mice during motivated epochs. **(A)** Eight natural scene images used during Stage 3 of change detection task (see Figure 2A). Number indicates label from De Vries et al., 2020. **(B)** Average response rates for all pairwise image transitions in the motivated state (averaged across all trials for each mouse, then across all mice in a genotype, and finally across genotypes). Catch trials (same-to-same transitions) lie along the diagonal. **(C)** Average response rate for each image-pair transition. *X*-axis is ordered by average response rate across all 64 transitions. Data is the same as shown in **(B)** with color values conserved. **(D)** Mean response rate for each image-pair, separated by genotype. The color for each image pair is conserved from **(B)** and the rank order is conserved from **(C)**. Gray points show response rate for each transition for each mouse in a given genotype. **(E)** In the motivated state, each genotype’s pattern of responding was strongly correlated with the average over all mice. **(F)** Response patterns in the motivated state are strongly correlated between all genotypes. Diagonal terms (with *p* = 1.0) and above diagonal terms (with values equal to the below diagonal terms) are excluded from the display. **(G)** Bootstrapped correlations of response patterns across genotypes are higher in the motivated compared to under- and over-motivated states. Absolute values are lower compared to **(F)** due to subsampling to match small trial counts in the over-motivated state.

In the final stage of training (stage 3), mice perform the visual change detection task with a set of 8 natural scene images (Figure 5A). In total, mice see 8 × 8 = 64 unique image-pair transitions (8 of these are no-change transitions, which define catch trials). On average, mice displayed a range of response probabilities to the 64 unique image pairs, indicating some transitions were more difficult than others (Figures 5B,C). Of the 56 mice with at least one expert session, four mice had fewer than an average of 4 presentations of each of the 64 possible natural image pairs (256 total trials) and were therefore excluded from these and subsequent analyses. The matrix shown in Figure 5B (and values plotted in Figure 5C) represents the grand average across all genotypes (an average matrix was computed for each mouse and then this was averaged over all mice). The pattern of behavioral responses across the set of image transitions reflects the mice’s perceptual landscape and this might differ across transgenic lines. Thus, we next sought to determine how similar was the pattern of behavioral responses across genotypes and whether this was motivation-dependent.

The rank order of the response probabilities for the 64 transitions were largely conserved across genotypes (Figure 5D), and each genotype’s pattern of behavioral responses correlated strongly with the average of all mice (Figure 5E; *r*-values of 0.93–0.99, *p*-values < 0.001). Moreover, each transgenic line strongly correlated with the others, indicated by significant pairwise correlations between all possible pairs (Figure 5F; *r*-values of 0.82–0.97, *p*-values < 0.001). To compare the strength of these correlations across the three motivational states, we performed a bootstrapping analysis to create response matrices on the subsampled data. Figure 5G shows the mean Pearson’s correlation coefficients for each pair of genotypes, calculated across all bootstrap iterations. We found that response correlations were highest in the optimally motivated state compared to over- and under-motivated states for all genotype combinations (Figure 5G, all *p*-values < 0.001).

## Discussion

We set out to characterize learning and behavioral performance of multiple transgenic mouse lines on a visual change detection task and to further understand how variation in motivation influences performance once trained. Overall, our results show that despite some differences in learning and motivation, the five GCaMP6 transgenic mouse lines we tested have highly correlated visual perception during optimally motivated states.

### Standardized Behavior Training of Transgenic Mice

An overarching goal of this work is to establish standardized training protocols to implement a robust behavior pipeline for characterization of cellular physiology using our *Allen Brain Observatory*. The transgenic lines we tested allow measurement of activity in specific subsets of excitatory cells (Cux2-CreERT2: Layers 2/3, Rbp4-Cre_KL100: Layer 5, Slc17a7-IRES2-Cre: Layers 1-6), and distinct inhibitory cell classes (Sst-IRES-Cre, Vip-IRES-Cre). As part of our development process it was important to anticipate experimental throughput by quantifying learning times and verifying robust task performance in these transgenic lines. Our results described here extend the basic phenotypic characterization of these transgenic lines (Daigle et al., 2018).

We trained all mice with an automated protocol that applied consistent parameters and task progression rules. All transgenic lines could be reliably trained in several weeks to perform the task using our protocol. Vip mice required significantly longer to reach the final stage of the task but performed at similar levels once trained. Additionally, although Sst mice learned the task quickly, they exhibited lower performance (d-prime) in the motivated state.

The underlying causes of the differences in motivation, learning, and performance in these transgenic lines is not apparent from this study. Follow-up work is necessary to determine whether these differences are related to potential disruption of neuronal activity by GCaMP6 expression. Alternatively, the cause could be due to developmental defects. For instance, developmental disruption of Vip interneurons is known to impair perceptual learning in mice (Batista-Brito et al., 2017). Another possibility is that the differences result from off-target effects on other brain or body systems in which GCaMP6 in non-selectively expressed in these transgenic lines. Consistent with this idea is the previous finding that Sst transgenic mice have an increased incidence of health-related issues including a propensity for dermatitis and this could impact their behavioral performance (Allen Institute for Brain Science, 2016). Differences in task training times have been noted in other transgenic lines such as Vgat-ChR2 mice (Resulaj et al., 2018), which express channelrhodopsin in inhibitory neurons. Importantly, in our study, despite these differences in learning and motivation, we found that perceptual decisions were very consistent across different lines when comparing matched motivational states.

### Motivation Is Non-stationary

Even in well-trained subjects, psychophysical performance can be non-stationary over a behavioral session, varying with motivation, attention, confusion, and other factors (Andermann et al., 2010; Carandini and Churchland, 2013; Mcginley et al., 2015a; Berditchevskaia et al., 2016). Tasks using water restriction, as in our study, are subject to motivational changes due to decreasing thirst as water is consumed during the session. Studies often only consider average performance over the session or restrict session duration to avoid major motivational changes. Here, all mice completed 1-h sessions, independent of mouse performance and experimenter intervention. Inspired by a recent study of motivation dynamics in mice performing a go/no-go task (Berditchevskaia et al., 2016), we use the signal detection theory metric, ‘criterion,’ to help categorize epochs in the session as over-motivated, motivated, and under-motivated. Parsing behavior sessions according to motivation level helps to compare behavior and physiology across mice and transgenic lines under more controlled conditions.

In most mice, motivation systematically decreased over each behavioral session. This likely represents a decrease in thirst-based motivation as water is consumed in the task. Consistent with this, we observed changes in licking behavior, including lick reaction time (lick latency) and consumption lick count (response vigor), which have been linked to motivational changes (Berditchevskaia et al., 2016). Interestingly, recent work suggests a brain-wide network is involved in thirst regulated motivation (Allen et al., 2019). Thus, characterizing changes in thirst-based motivation will likely be important for interpreting neural activity measurements in tasks involving water reward.

We used a metric from signal detection theory, ‘criterion’ (Green and Swets, 1966), to estimate the motivational states of mice in our task. Future work can develop improved methods for identifying and quantifying behavioral states including generalized linear models and hidden Markov models (Wiltschko et al., 2015; Calhoun et al., 2019). These methods have the potential to provide a more powerful description of motivation, task-engagement, and other latent variables, and might also reduce the need for the temporal binning approach used here. In addition, they could help to explore how task contingencies and reinforcement structures affect motivation state and could provide insight into the factors that shape task learning, behavioral strategy, and ultimate performance levels.

It will be important in future work to relate motivation to other behavioral and physiological states. Pupillometry measurements can reflect internal states including levels of arousal and task-engagement (Mcginley et al., 2015b; Vinck et al., 2015). In addition, animal movements, including spontaneous actions and fidgets (Musall et al., 2019; Stringer et al., 2019), can be captured with whole body or face cameras and analysis of these behavioral data streams might provide additional quantitative correlates of motivation.

### Similar Perception Across Transgenic Mice

We used our behavioral task to assess natural image change detection in transgenic mice. Expert mice can differentiate each of the unique combinations of natural images tested, although some image pair transitions are more difficult to distinguish than others, consistent with a target/distractor paradigm in mice (Yu et al., 2018). The mouse lines we tested here show correlated behavioral responses, and this correlation is very high when mice are compared under matched motivation states. Thus, these transgenic lines show similar patterns of perception despite some differences in learning rates, motivation dynamics, and d-prime values.

In forthcoming physiological experiments, we will measure neural activity in these mice to characterize cellular correlates of change perception, task-engagement, short-term working memory, and temporal expectation. In an initial study of layer 2/3 excitatory and Vip inhibitory cells in visual cortex, we found that excitatory cells provide selective image coding in the task, whereas Vip cells undergo dramatic changes in activity dynamics with learning (Garrett et al., 2020). Large-scale systematic mapping of activity in different cell classes across the brain will provide insights into how these interactions mediate neural processing to guide behavior and learning.

## Data Availability Statement

The datasets generated for this study are available on request to the corresponding author.

## Ethics Statement

The animal study was reviewed and approved by Allen Institute Institutional Animal Care and Use Committee.

## Author Contributions

SO, PG, DO, SMa, SMi, JK, and MG: conceptualization. SO and PG: supervision. LC, SC, PG, KM, and JS: data collection. DO, SO, PG, SMa, SMi, JK, and MG: investigation, validation, methodology, and formal analyses. DW, DO, and JK: software. DO and JK: data curation. DO, PG, and JK: visualization. SO, PG, and DO: writing original draft with input from JK. All co-authors: manuscript review.

## Conflict of Interest

The authors declare that the research was conducted in the absence of any commercial or financial relationships that could be construed as a potential conflict of interest.

## Footnotes

1 http://observatory.brain-map.org/visualcoding

## References

[B1] Allen W. E. Chen M. Z. Pichamoorthy N. Tien R. H. Pachitariu M. Luo L. (2019). Thirst regulates motivated behavior through modulation of brainwide neural population dynamics. *Science* 364:eaav3932. 10.1126/science.aav3932 PMC6711472 30948440

[B2] Allen Institute for Brain Science K (2016). *Phenotypic Characterization of Transgenic Mouse Lines White Paper.* Seattle, WAı: Allen Institute, 1–17

[B3] Andermann M. L. Kerlin A. M. Reid R. C. (2010). Chronic cellular imaging of mouse visual cortex during operant behavior and passive viewing. *Front. Cell. Neurosci.* 4:3. 10.3389/fncel.2010.00003 PMC2854571 20407583

[B4] Batista-Brito R. Vinck M. Ferguson K. A. Chang J. T. Laubender D. Lur G. (2017). Developmental dysfunction of vip interneurons impairs cortical circuits. *Neuron* 95 884–895.e9. 10.1016/j.neuron.2017.07.034 28817803 PMC5595250

[B5] Berditchevskaia A. Cazé R. D. Schultz S. R. (2016). Performance in a GO/NOGO perceptual task reflects a balance between impulsive and instrumental components of behaviour. *Sci. Rep.* 6:27389. 10.1038/srep27389 PMC4895381 27272438

[B6] Brunet N. M. Bosman C. A. Vinck M. Roberts M. Oostenveld R. Desimone R. (2014). Stimulus repetition modulates gamma-band synchronization in primate visual cortex. *Proc. Natl. Acad. Sci. U.S.A.* 111 3626–3631. 10.1073/pnas.1309714111 24554080 PMC3948273

[B7] Burgess C. P. Lak A. Steinmetz N. A. Zatka-Haas P. Bai Reddy C. Jacobs E. A. K. (2017). High-yield methods for accurate two-alternative visual psychophysics in head-fixed mice. *Cell Rep.* 20 2513–2524. 10.1016/j.celrep.2017.08.047 28877482 PMC5603732

[B8] Calhoun A. J. Pillow J. W. Murthy M. (2019). Unsupervised identification of the internal states that shape natural behavior that shape natural behavior. *Nat. Neurosci.* 22 2040–2049. 10.1038/s41593-019-0533-x 31768056 PMC7819718

[B9] Carandini M. Churchland A. K. (2013). Probing perceptual decisions in rodents. *Nat. Neurosci.* 16 824–831. 10.1038/nn.3410 23799475 PMC4105200

[B10] Chen J. L. Carta S. Soldado-Magraner J. Schneider B. L. Helmchen F. (2013). Behaviour-dependent recruitment of long-range projection neurons in somatosensory cortex. *Nature* 499 336–340. 10.1038/nature12236 23792559

[B11] Daigle T. L. Madisen L. Hage T. A. Valley M. T. Knoblich U. Larsen R. S. (2018). A suite of transgenic driver and reporter mouse lines with enhanced brain-cell-type targeting and functionality. *Cell* 174 465–480.e22. 10.1016/j.cell.2018.06.035 30007418 PMC6086366

[B12] De Vries S. E. J. Lecoq J. A. Buice M. A. Groblewski P. A. Ocker G. K. Oliver M. (2020). A large-scale standardized physiological survey reveals functional organization of the mouse visual cortex. *Nat. Neurosci.* 23 138–151. 31844315 10.1038/s41593-019-0550-9 PMC6948932

[B13] Denman D. J. Luviano J. A. Ollerenshaw D. R. Cross S. Williams D. Buice M. A. (2018). Mouse color and wavelength-specific luminance contrast sensitivity are non- uniform across visual space. *Elife* 7 1–16. 10.7554/eLife.31209 PMC5762155 29319502

[B14] Duffy E. (1957). The psychological significance of the concept of “arousal” or “activation.”. *Psychol. Rev.* 64 265–275. 10.1037/h0048837 13494613

[B15] Elmore L. C. Ji, Ma W. Magnotti J. F. Leising K. J. Passaro A. D. (2011). Visual short-term memory compared in rhesus monkeys and humans. *Curr. Biol.* 21 975–979. 10.1016/j.cub.2011.04.031 21596568 PMC4634532

[B16] Garrett M. E. Manavi S. Roll K. Ollerenshaw D. R. Groblewski P. A. Kiggins J. (2020). Experience shapes activity dynamics and stimulus coding of VIP inhibitory and excitatory cells in visual cortex. *Elife* 9:e50340. 32101169 10.7554/eLife.50340 PMC7043888

[B17] Glickfeld L. L. Histed M. H. Maunsell J. H. R. (2013). Mouse primary visual cortex is used to detect both orientation and contrast changes. *J. Neurosci.* 33 19416–19422. 10.1523/jneurosci.3560-13.2013 24336708 PMC3858618

[B18] Goard M. J. Pho G. N. Woodson J. Sur M. (2016). Distinct roles of visual, parietal, and frontal motor cortices in memory-guided sensorimotor decisions. *Elife* 5:e13764. 10.7554/eLife.13764 PMC4974053 27490481

[B19] Green D. M. Swets J. A. (1966). *Signal detection Theory and Psychophysics.* New York, NY: Wiley.

[B20] Groblewski P. Sullivan D. Lecoq J. de Vries S. Caldejon S. L’Heureux Q. (2020a). A standardized head-fixation system for performing large-scale, in-vivo physiological recordings in mice. *BioRxiv.* 10.1101/2020.01.22.916007 32946912

[B21] Groblewski P. A. Ollerenshaw D. R. Kiggins J. Garrett M. (2020b). Similar visual perception in GCaMP6 transgenic mice despite differences in learning and motivation. *BioRxiv.* 10.1101/2020.02.18.954990

[B22] Guo Z. Li N. Huber D. Ophir E. Gutnisky D. Ting J. (2014a). Flow of cortical activity underlying a tactile decision in mice. *Neuron* 81 179–194. 10.1016/j.neuron.2013.10.020 24361077 PMC3984938

[B23] Guo Z. V. Hires S. A. Li N. O’Connor D. H. Komiyama T. Ophir E. (2014b). Procedures for Behavioral Experiments in Head-Fixed Mice. *PLoS One* 9:e88678. 10.1371/journal.pone.0088678 24520413 PMC3919818

[B24] Hagmann C. E. Cook R. G. (2013). Active change detection by pigeons and humans. *J. Exp. Psychol. Anim. Behav. Process.* 39 383–389. 10.1037/a0033313 23875530 PMC4129511

[B25] Harris K. D. Shepherd G. M. G. G. (2015). The neocortical circuit: themes and variations. *Nat. Neurosci.* 18 170–181. 10.1038/nn.3917 25622573 PMC4889215

[B26] Harvey C. D. Coen P. Tank D. W. (2012). Choice-specific sequences in parietal cortex during a virtual-navigation decision task. *Nature* 484 62–68. 10.1038/nature10918 22419153 PMC3321074

[B27] Histed M. H. Carvalho L. A. Maunsell J. H. R. (2012). Psychophysical measurement of contrast sensitivity in the behaving mouse. *J. Neurophysiol.* 107 758–765. 10.1152/jn.00609.2011 22049334 PMC3289478

[B28] Hu B. Garrett M. E. Groblewski P. A. Ollerenshaw D. R. Shang J. Roll K. (2020). Adaptation supports short-term memory in a visual change detection task. *BioRxiv.* 10.1101/2020.03.06.977512 PMC8480767 34534203

[B29] Huber D. Gutnisky D. A. Peron S. O’Connor D. H. Wiegert J. S. Tian L. (2012). Multiple dynamic representations in the motor cortex during sensorimotor learning. *Nature* 484 473–478. 10.1038/nature11039 22538608 PMC4601999

[B30] Kepecs A. Fishell G. (2014). Interneuron cell types are fit to function. *Nature* 505 318–326. 10.1038/nature12983 24429630 PMC4349583

[B31] Koch C. Reid R. C. (2012). Neuroscience: observatories of the mind. *Nature* 483:397. 10.1038/483397a 22437592

[B32] Li N. Daie K. Svoboda K. Druckmann S. (2016). Robust neuronal dynamics in premotor cortex during motor planning. *Nature* 532 459–464. 10.1038/nature17643 27074502 PMC5081260

[B33] Luo L. Callaway E. M. Svoboda K. (2008). Genetic dissection of neural circuits. *Neuron* 57 634–660. 18341986 10.1016/j.neuron.2008.01.002 PMC2628815

[B34] Luo L. Callaway E. M. Svoboda K. (2018). Genetic dissection of neural circuits: a decade of progress. *Neuron* 98 256–281. 10.1016/j.neuron.2018.03.040 29673479 PMC5912347

[B35] Macmillan N. A. Creelman C. D. (2004). *Detection Theory: A user’s Guide.* Hove: Psychology press.

[B36] Madisen L. Garner A. R. Shimaoka D. Chuong A. S. Klapoetke N. C. Li L. (2015). Transgenic mice for intersectional targeting of neural sensors and effectors with high specificity and performance. *Neuron* 85 942–958. 10.1016/j.neuron.2015.02.022 25741722 PMC4365051

[B37] Mcginley M. J. David S. V. Mccormick D. A. (2015a). Cortical membrane potential signature of optimal states for sensory signal detection. *Neuron* 87 179–192. 10.1016/j.neuron.2015.05.038 26074005 PMC4631312

[B38] Mcginley M. J. Vinck M. Reimer J. Batista-Brito R. Zagha E. Cadwell C. R. (2015b). Waking state: rapid variations modulate neural and behavioral responses. *Neuron* 87 1143–1161. 10.1016/j.neuron.2015.09.012 26402600 PMC4718218

[B39] Musall S. Kaufman M. T. Juavinett A. L. Gluf S. Churchland A. K. (2019). Single-trial neural dynamics are dominated by richly varied movements. *Nat. Neurosci.* 22 1677–1686. 10.1038/s41593-019-0502-4 31551604 PMC6768091

[B40] Niell C. M. (2015). Cell types, circuits, and receptive fields in the mouse visual cortex. *Annu. Rev. Neurosci.* 38 413–431. 10.1146/annurev-neuro-071714-033807 25938727

[B41] O’Connor D. H. Clack N. G. Huber D. Komiyama T. Myers E. W. Svoboda K. (2010). Vibrissa-based object localization in head-fixed mice. *J. Neurosci.* 30 1947–1967. 10.1523/jneurosci.3762-09.2010 20130203 PMC6634009

[B42] O’Connor D. H. Hires S. A. Guo Z. V. Li N. Yu J. Sun Q.-Q. (2013). Neural coding during active somatosensation revealed using illusory touch. *Nat. Neurosci.* 16 958–965. 10.1038/nn.3419 23727820 PMC3695000

[B43] Pearson J. M. Platt M. L. (2013). Change detection, multiple controllers, and dynamic environments: Insights from the brain. *J. Exp. Anal. Behav.* 99 74–84. 10.1002/jeab.5 23344989 PMC4153983

[B44] Peron S. P. Freeman J. Guo C. Svoboda K. Peron S. P. Freeman J. (2015). A cellular resolution map of barrel cortex activity during tactile behavior. *Neuron* 86 783–799. 10.1016/j.neuron.2015.03.027 25913859

[B45] Petreanu L. Gutnisky D. A. Huber D. Xu N. O’Connor D. H. Tian L. (2012). Activity in motor-sensory projections reveals distributed coding in somatosensation. *Nature* 489 299–303. 10.1038/nature11321 22922646 PMC3443316

[B46] Pinto L. Goard M. J. Estandian D. Xu M. Kwan A. C. Lee S.-H. (2013). Fast modulation of visual perception by basal forebrain cholinergic neurons. *Nat. Neurosci.* 16 1857–1863. 10.1038/nn.3552 24162654 PMC4201942

[B47] Poort J. Khan A. G. G. Pachitariu M. Nemri A. Orsolic I. Krupic J. (2015). Learning enhances sensory and multiple non-sensory representations in primary visual cortex. *Neuron* 86 1478–1490. 10.1016/j.neuron.2015.05.037 26051421 PMC4503798

[B48] Rensink R. A. (2002). Change detection. *Annu. Rev. Psychol.* 53 245–277. 11752486 10.1146/annurev.psych.53.100901.135125

[B49] Resulaj A. Ruediger S. Olsen S. R. Scanziani M. (2018). First spikes in visual cortex enable perceptual discrimination. *Elife* 7 1–22. 10.7554/eLife.34044 PMC5902162 29659352

[B50] Saravanan V. Berman G. J. Sober S. J. (2019). Application of the hierarchical bootstrap to multi-level data in neuroscience. *BioRxiv.* [Preprint]. 819334. PMC7906290 33644783

[B51] Stringer C. Pachitariu M. Steinmetz N. Reddy C. B. Carandini M. Harris K. D. (2019). Spontaneous behaviors drive multidimensional, brainwide activity. *Science* 364:255. 10.1126/science.aav7893 PMC6525101 31000656

[B52] Vinck M. Batista-Brito R. Knoblich U. Cardin J. A. (2015). Arousal and locomotion make distinct contributions to cortical activity patterns and visual encoding. *Neuron* 86, 740–754. 10.1016/j.neuron.2015.03.028 25892300 PMC4425590

[B53] Wiltschko A. B. Johnson M. J. Iurilli G. Peterson R. E. Katon J. M. Pashkovski S. L. (2015). Mapping sub-second structure in mouse behavior. *Neuron* 88 1121–1135. 10.1016/j.neuron.2015.11.031 26687221 PMC4708087

[B54] Womelsdorf T. Fries P. Mitra P. P. Desimone R. (2006). Gamma-band synchronization in visual cortex predicts speed of change detection. *Nature* 439 733–736. 10.1038/nature04258 16372022

[B55] Yerkes R. M. Dodson J. D. (1908). The relation of strength of stimulus to rapidity of habit-formation. *J. Comp. Neurol. Psychol.* 18 459–482. 10.1002/cne.920180503

[B56] Yu Y. Hira R. Stirman J. N. Yu W. Smith I. T. Smith S. L. (2018). Mice use robust and common strategies to discriminate natural scenes. *Sci. Rep.* 8:1379. 10.1038/s41598-017-19108-w PMC5778028 29358739

