# Supplementary material for: Characterization of Learning, Motivation, and Visual Perception in Five Transgenic Mouse Lines Expressing GCaMP in Distinct Cell Populations
Source: Front Behav Neurosci. 2020 Jun 23;14:104. doi: 10.3389/fnbeh.2020.00104 (PMC7324787; doi:10.3389/fnbeh.2020.00104)
Supplement: Supplementary file 1 [file Data_Sheet_1.docx]

Supplementary Material


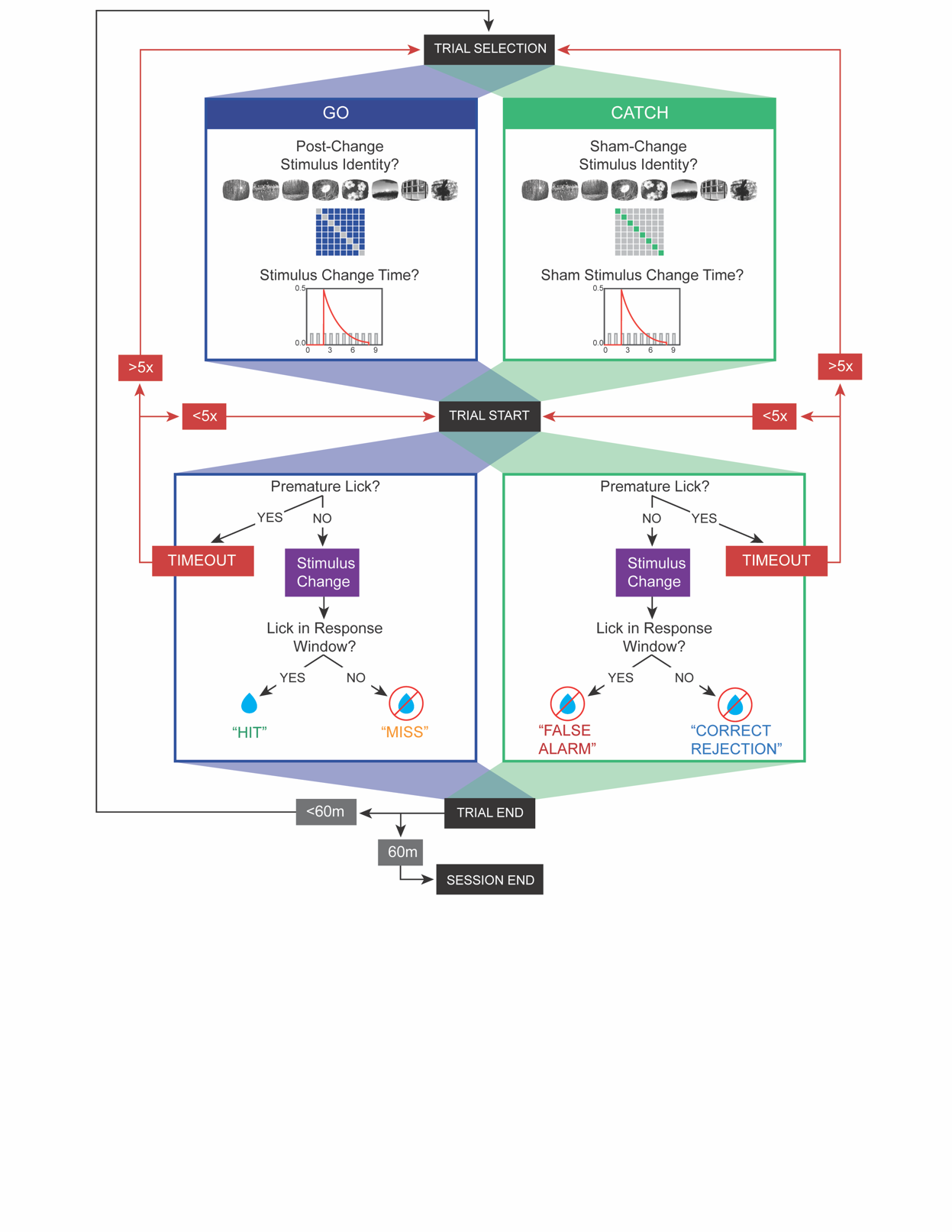


**Supplemental Figure 1. Task flow diagram.**

The “Flashed Images” stage of the change detection task consists of 8 images, resulting in 64 possible image transitions, including both GO and CATCH trials. GO trials comprise 87.5% of all trials and are represented in the off-diagonal portions of the 8x8 change matrix. CATCH trials comprise 12.5% of all trials and are represented in the diagonal of the matrix. Each trial was first selected as either GO or CATCH and a post-change (or sham-change) image identity was chosen from the change matrix. The stimulus change (or sham-change) time was then selected from a truncated exponential distribution between 2.25s to 8.25s. As stimuli are presented every 715 ms, the actual change time was determined as the nearest flash from the drawn time. Once a trial started, a premature lick (i.e., a lick that occurred prior to the predetermined change time) resulted in a timeout and the trial was restarted. If an animal caused a trial to timeout 5 times, a new trial was selected. If no premature licks were recorded, the trial progressed and the stimulus change occurred at the predetermined change-time. On GO trials, a lick detected within 600ms response window resulted in a “HIT” (and subsequent reward delivered) whereas a lack of response resulted in a “MISS”. On CATCH trials, a lick within the window following the sham-change resulted in a “FALSE ALARM”, whereas a lack of response resulted in a “CORRECT REJECTION”. Following the stimulus change and response window the trial ended and a new trial was selected. The session ended after 60 minutes.


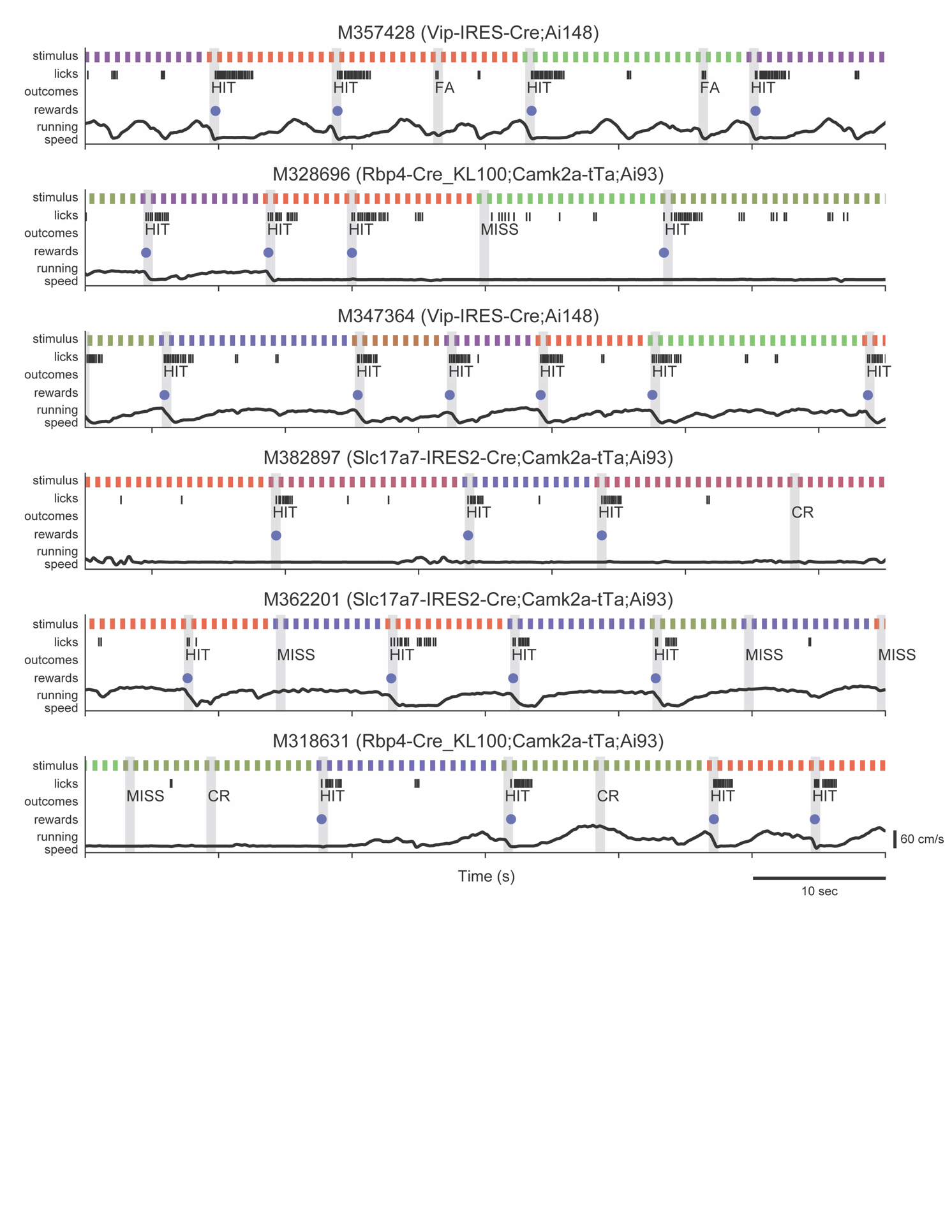


**Supplemental Figure 2. Example behavioral segments.**

One-minute examples of behavior from 6 mice of various genotypes. Each example shows stimulus (color-coded by image identity to show when changes occur), licks, trial outcome, reward delivery, and running speed.

**
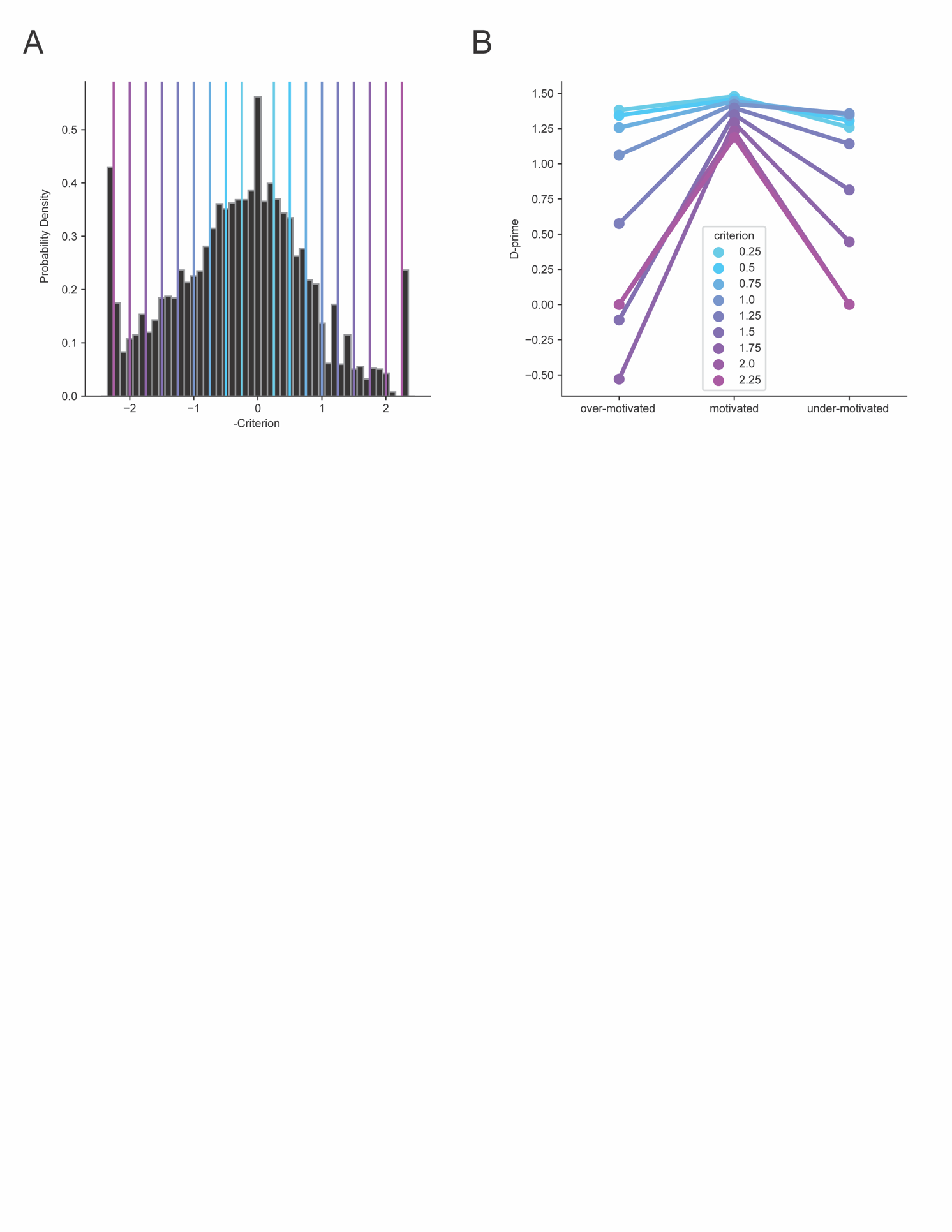
**

**Supplemental Figure 3. D-prime in each motivation state for a range of criterion thresholds.**

**A)** The histogram of criterion values (same as Fig 3E) with a range of criterion thresholds drawn. Thresholds range from +/- 0.5 to +/- 2.25 in increments of 0.25. In every case, the ‘motivated’ epochs are designated as those that fall between the thresholds, the over motivated epochs are those that fall to the right of the higher threshold and the under motivated epochs are those that fall to the left of the lower threshold. Note that thresholds of +/- 1.25 were used in the main figures. **B)** D-prime calculated on all pooled trials in each of the three motivation states for the range of criterion values shown in A.

# Table 1. Mice included in study.

| **mouse_id** | **genotype** | **sex** | **age (days) at start of training** | **days in stage 1** | **days in stage 2** | **days in stage 3** | **analyzed stage 3 sessions** | **# motivated trials** | **# under-motivated trials** | **# over-motivated trials** | **# un-categorized trials** |
| --- | --- | --- | --- | --- | --- | --- | --- | --- | --- | --- | --- |
| **M373115** | Cux2-CreERT2 | M | 79 | 4 | 1 | 29 | 24 | 2634 | 663 | 705 | 95 |
| **M373118** | Cux2-CreERT2 | M | 118 | 3 | 1 | 37 | 36 | 9140 | 144 | 462 | 6 |
| **M374446** | Cux2-CreERT2 | M | 111 | 2 | 1 | 38 | 37 | 7190 | 1641 | 608 | 15 |
| **M389532** | Cux2-CreERT2 | M | 95 | 3 | 1 | 37 | 37 | 6213 | 1742 | 718 | 47 |
| **M318631** | Rbp4-Cre_KL100 | M | 102 | 5 | 3 | 10 | 9 | 2505 | 431 | 0 | 0 |
| **M318635** | Rbp4-Cre_KL100 | F | 102 | 3 | 1 | 16 | 13 | 3566 | 402 | 0 | 0 |
| **M328341** | Rbp4-Cre_KL100 | M | 77 | 4 | 2 | 24 | 21 | 4051 | 1657 | 43 | 0 |
| **M328344** | Rbp4-Cre_KL100 | F | 77 | 2 | 1 | 38 | 34 | 8953 | 4574 | 0 | 0 |
| **M328696** | Rbp4-Cre_KL100 | M | 76 | 3 | 1 | 37 | 36 | 9968 | 103 | 102 | 3 |
| **M328933** | Rbp4-Cre_KL100 | F | 75 | 3 | 1 | 37 | 28 | 7109 | 1356 | 0 | 1 |
| **M330194** | Rbp4-Cre_KL100 | M | 84 | 3 | 1 | 37 | 24 | 5817 | 333 | 11 | 0 |
| **M330196** | Rbp4-Cre_KL100 | F | 84 | 3 | 2 | 36 | 35 | 8866 | 4008 | 0 | 0 |
| **M348627** | Rbp4-Cre_KL100 | F | 81 | 5 | 2 | 27 | 21 | 3407 | 2659 | 76 | 20 |
| **M370023** | Rbp4-Cre_KL100 | M | 135 | 4 | 1 | 26 | 24 | 3184 | 68 | 461 | 77 |
| **M376756** | Rbp4-Cre_KL100 | F | 100 | 3 | 1 | 25 | 16 | 2385 | 2221 | 15 | 23 |
| **M376801** | Rbp4-Cre_KL100 | F | 134 | 4 | 1 | 0 | 0 | 0 | 0 | 0 | 0 |
| **M324022** | Slc17a7-IRES2-Cre | M | 81 | 9 | 2 | 29 | 21 | 4023 | 1186 | 55 | 0 |
| **M324023** | Slc17a7-IRES2-Cre | M | 81 | 12 | 4 | 25 | 12 | 3063 | 577 | 0 | 0 |
| **M324030** | Slc17a7-IRES2-Cre | F | 81 | 5 | 4 | 11 | 11 | 2298 | 1176 | 0 | 0 |
| **M333706** | Slc17a7-IRES2-Cre | F | 69 | 2 | 1 | 38 | 33 | 6563 | 1698 | 233 | 4 |
| **M334310** | Slc17a7-IRES2-Cre | F | 66 | 4 | 1 | 35 | 35 | 8961 | 1215 | 71 | 0 |
| **M336340** | Slc17a7-IRES2-Cre | M | 58 | 6 | 1 | 34 | 34 | 8989 | 1918 | 0 | 0 |
| **M336349** | Slc17a7-IRES2-Cre | F | 58 | 11 | 1 | 29 | 24 | 5095 | 829 | 120 | 7 |
| **M347745** | Slc17a7-IRES2-Cre | M | 86 | 4 | 1 | 36 | 34 | 8005 | 714 | 176 | 0 |
| **M355469** | Slc17a7-IRES2-Cre | M | 72 | 11 | 1 | 29 | 28 | 5605 | 3749 | 115 | 0 |
| **M354477** | Slc17a7-IRES2-Cre | F | 98 | 3 | 4 | 34 | 30 | 5259 | 2201 | 205 | 0 |
| **M355471** | Slc17a7-IRES2-Cre | M | 94 | 4 | 1 | 25 | 25 | 5053 | 2149 | 164 | 30 |
| **M362197** | Slc17a7-IRES2-Cre | M | 91 | 3 | 1 | 24 | 22 | 3203 | 667 | 376 | 42 |
| **M362201** | Slc17a7-IRES2-Cre | M | 92 | 3 | 1 | 19 | 19 | 3463 | 233 | 411 | 38 |
| **M363140** | Slc17a7-IRES2-Cre | M | 102 | 3 | 8 | 30 | 26 | 5885 | 1736 | 149 | 12 |
| **M369320** | Slc17a7-IRES2-Cre | M | 87 | 2 | 2 | 33 | 32 | 4114 | 4482 | 469 | 8 |
| **M369315** | Slc17a7-IRES2-Cre | F | 88 | 5 | 2 | 10 | 7 | 769 | 509 | 81 | 0 |
| **M369578** | Slc17a7-IRES2-Cre | F | 86 | 2 | 1 | 14 | 14 | 2147 | 1886 | 92 | 28 |
| **M382895** | Slc17a7-IRES2-Cre | F | 98 | 3 | 1 | 1 | 1 | 278 | 0 | 0 | 0 |
| **M382897** | Slc17a7-IRES2-Cre | F | 98 | 3 | 3 | 35 | 35 | 6146 | 1092 | 716 | 7 |
| **M385255** | Slc17a7-IRES2-Cre | M | 86 | 3 | 1 | 15 | 14 | 2075 | 809 | 255 | 31 |
| **M384942** | Slc17a7-IRES2-Cre | F | 95 | 3 | 1 | 14 | 14 | 1687 | 2304 | 109 | 7 |
| **M387109** | Slc17a7-IRES2-Cre | F | 89 | 3 | 1 | 34 | 28 | 6333 | 1689 | 78 | 0 |
| **M390905** | Slc17a7-IRES2-Cre | M | 88 | 4 | 1 | 2 | 2 | 154 | 198 | 55 | 20 |
| **M358210** | Sst-IRES-Cre | M | 79 | 6 | 1 | 34 | 21 | 1794 | 1047 | 325 | 130 |
| **M358211** | Sst-IRES-Cre | M | 113 | 3 | 4 | 22 | 11 | 806 | 0 | 398 | 41 |
| **M360543** | Sst-IRES-Cre | M | 100 | 4 | 25 | 0 | 0 | 0 | 0 | 0 | 0 |
| **M358213** | Sst-IRES-Cre | M | 114 | 2 | 2 | 15 | 5 | 398 | 239 | 93 | 42 |
| **M358809** | Sst-IRES-Cre | M | 110 | 5 | 6 | 6 | 4 | 503 | 717 | 23 | 0 |
| **M366616** | Sst-IRES-Cre | M | 102 | 3 | 1 | 12 | 2 | 157 | 419 | 14 | 0 |
| **M389575** | Sst-IRES-Cre | M | 81 | 4 | 2 | 30 | 9 | 1068 | 457 | 122 | 5 |
| **M327444** | Vip-IRES-Cre | F | 81 | 5 | 9 | 27 | 1 | 144 | 0 | 0 | 0 |
| **M330982** | Vip-IRES-Cre | M | 67 | 13 | 26 | 2 | 2 | 314 | 462 | 0 | 0 |
| **M329069** | Vip-IRES-Cre | F | 88 | 22 | 16 | 3 | 3 | 479 | 582 | 0 | 0 |
| **M329071** | Vip-IRES-Cre | F | 88 | 13 | 1 | 27 | 24 | 3822 | 3039 | 48 | 0 |
| **M333115** | Vip-IRES-Cre | F | 72 | 32 | 7 | 0 | 0 | 0 | 0 | 0 | 0 |
| **M333117** | Vip-IRES-Cre | F | 72 | 10 | 2 | 4 | 2 | 139 | 127 | 15 | 14 |
| **M336247** | Vip-IRES-Cre | F | 58 | 7 | 0 | 0 | 0 | 0 | 0 | 0 | 0 |
| **M347364** | Vip-IRES-Cre | M | 104 | 5 | 4 | 31 | 28 | 3745 | 5018 | 107 | 2 |
| **M357428** | Vip-IRES-Cre | M | 83 | 10 | 14 | 17 | 15 | 1969 | 3174 | 80 | 0 |
| **M363894** | Vip-IRES-Cre | F | 81 | 3 | 1 | 24 | 23 | 2282 | 3098 | 282 | 50 |
| **M363887** | Vip-IRES-Cre | M | 82 | 11 | 15 | 15 | 15 | 2286 | 1439 | 176 | 8 |
| **M365869** | Vip-IRES-Cre | M | 89 | 6 | 13 | 20 | 20 | 2174 | 2974 | 403 | 0 |
| **M363890** | Vip-IRES-Cre | F | 103 | 11 | 1 | 7 | 6 | 795 | 842 | 37 | 0 |
| **M385947** | Vip-IRES-Cre | M | 102 | 5 | 12 | 13 | 13 | 2109 | 826 | 128 | 4 |

**
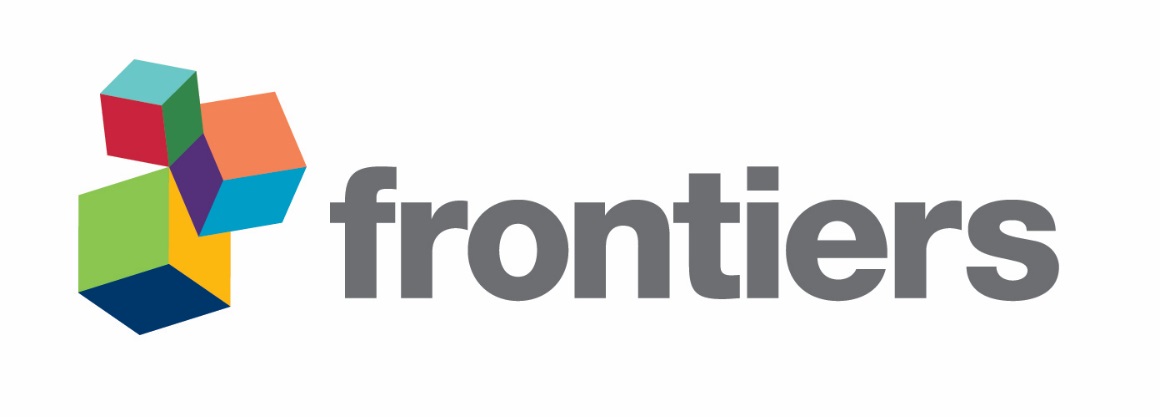
**
